# Supplementary material for: Rewritable two-dimensional DNA-based data storage with machine learning reconstruction
Source: Nat Commun. 2022 May 27;13:2984. doi: 10.1038/s41467-022-30140-x (PMC9142566; doi:10.1038/s41467-022-30140-x)
Supplement: Supplementary file 2 — Reporting Summary [file 41467_2022_30140_MOESM2_ESM.pdf]

nature research

Corresponding author(s): Charles M Schroeder, Olgica Milenkovic

Last updated by author(s): Dec 13, 2021

Reporting Summary

Nature Research wishes to improve the reproducibility of the work that we publish. This form provides structure for consistency and transparency in reporting. For further information on Nature Research policies, see our Editorial Policies and the Editorial Policy Checklist.

Statistics

For all statistical analyses, confirm that the following items are present in the figure legend, table legend, main text, or Methods section.

n/a	Confirmed	
☐	☒	The exact sample size (*n*) for each experimental group/condition, given as a discrete number and unit of measurement
☐	☒	A statement on whether measurements were taken from distinct samples or whether the same sample was measured repeatedly
☒	☐	The statistical test(s) used AND whether they are one- or two-sided<br>*Only common tests should be described solely by name; describe more complex techniques in the Methods section.*
☒	☐	A description of all covariates tested
☐	☒	A description of any assumptions or corrections, such as tests of normality and adjustment for multiple comparisons
☒	☐	A full description of the statistical parameters including central tendency (e.g. means) or other basic estimates (e.g. regression coefficient) AND variation (e.g. standard deviation) or associated estimates of uncertainty (e.g. confidence intervals)
☒	☐	For null hypothesis testing, the test statistic (e.g. *F*, *t*, *r*) with confidence intervals, effect sizes, degrees of freedom and *P* value noted<br>*Give P values as exact values whenever suitable.*
☒	☐	For Bayesian analysis, information on the choice of priors and Markov chain Monte Carlo settings
☒	☐	For hierarchical and complex designs, identification of the appropriate level for tests and full reporting of outcomes
☒	☐	Estimates of effect sizes (e.g. Cohen's *d*, Pearson's *r*), indicating how they were calculated

Our web collection on statistics for biologists contains articles on many of the points above.

Software and code

Policy information about availability of computer code

Data collection	All sequencing experiments were performed by MiSeq (Illumina, CA) for 250 cycles with a Nano V2 500 cycles kit (Illumina). The raw fastq files were generated and demultiplexed with the bcl2fastq v2.20 Conversion Software (Illumina).
Data analysis	SnapGene Viewer 5.1.7 was used for visualizing the DNA sequence and nicking enzyme positions. All raw fastq files were generated and demultiplexed with the bcl2fastq v2.20 Conversion Software (Illumina). The custom codes used in this study were written in Python and are available at https://github.com/thupchnsky/2DDNA.

For manuscripts utilizing custom algorithms or software that are central to the research but not yet described in published literature, software must be made available to editors and reviewers. We strongly encourage code deposition in a community repository (e.g. GitHub). See the Nature Research guidelines for submitting code & software for further information.

Data

Policy information about availability of data

All manuscripts must include a data availability statement. This statement should provide the following information, where applicable:

- Accession codes, unique identifiers, or web links for publicly available datasets
- A list of figures that have associated raw data
- A description of any restrictions on data availability

All sequencing data are accessible at: https://doi.org/10.13012/B2IDB-2308557_V1. All custom algorithms used are listed and described at https://github.com/thupchnsky/2DDNA.

Field-specific reporting

Please select the one below that is the best fit for your research. If you are not sure, read the appropriate sections before making your selection.

☒ Life sciences ☐ Behavioural & social sciences ☐ Ecological, evolutionary & environmental sciences

For a reference copy of the document with all sections, see nature.com/documents/nr-reporting-summary-flat.pdf

Life sciences study design

All studies must disclose on these points even when the disclosure is negative.

Sample size	The stored files were chosen arbitrarily. The sizes were selected in the range of kbytes in order to keep the implementation cost at a reasonable level
Data exclusions	No data was excluded, all the experimental/analysis outputs are represented in the manuscript or supplementary information.
Replication	All findings are effectively reproducible. Reproducibility was established by repeating all the writing/erasing/rewriting/reading experiments 3 or more times, testing the method on different files,DNA sequences, enzymes and buffers.
Randomization	In our case no randomization was needed, all samples were treated equally.
Blinding	The researchers were blind to the sequencing results before decoding procedure, and all consensus sequences were obtained by alignment algorithms. During decoding procedure of the first storage dimension (image content), specific encoding setup (length of oligos, Hilbert space-filling curves and Huffman dictionary generated in encoding procedure, etc.) was assumed to be known. During decoding procedure of the second storage dimension (image metadata), the researchers were blind to the reference sequences, and the method to obtain approximate reference sequences is introduced in the main text.

Reporting for specific materials, systems and methods

We require information from authors about some types of materials, experimental systems and methods used in many studies. Here, indicate whether each material, system or method listed is relevant to your study. If you are not sure if a list item applies to your research, read the appropriate section before selecting a response.

Materials & experimental systems

n/a	Involved in the study	
☒	☐	Antibodies
☒	☐	Eukaryotic cell lines
☒	☐	Palaeontology and archaeology
☒	☐	Animals and other organisms
☒	☐	Human research participants
☒	☐	Clinical data
☒	☐	Dual use research of concern

Methods

n/a	Involved in the study	
☒	☐	ChIP-seq
☒	☐	Flow cytometry
☒	☐	MRI-based neuroimaging
